# *Candida haemulonii* Complex Species, Brazil, January 2010–March 2015

**DOI:** 10.3201/eid2203.151610

**Published:** 2016-03

**Authors:** João Nobrega de Almeida, João Guilherme Pontes Lima Assy, Anna S. Levin, Gilda M.B. Del Negro, Mauro C. Giudice, Marcela Pullice Tringoni, Danilo Yamamoto Thomaz, Adriana Lopes Motta, Edson Abdala, Ligia Camara Pierroti, Tania Strabelli, Ana Lucia Munhoz, Flávia Rossi, Gil Benard

**Affiliations:** Universidade de São Paulo, São Paulo, Brazil

**Keywords:** Candida, C. haemulonii, C. haemulonii var. vulnera, C. duobushaemulonii, fungal infections, fungi, fungemia, candidiasis, yeast, antifungal susceptibility, Staphylococcus, amphotericin B, fluconazole, voriconazole, caspofungin, anidulafungin, Brazil

**To the Editor:** The epidemiology of yeast infections is evolving, and species in the *Candida haemulonii* complex have been identified as a cause of candidiasis (*1*). In 2012, *C. haemulonii* complex was reclassified as 2 species and 1 variety: *C. haemulonii* (former group I), *C. duobushaemulonii* (former group II) and *C. haemulonii* var. *vulnera* (*1*).

Despite the growing knowledge about the biology and clinical relevance of these pathogens, species-specific data comparing clinical and microbiological aspects are lacking. We describe the clinical and microbiological characteristics of patients from 5 hospitals in São Paulo, Brazil, whose cultures were positive for the *C. haemulonii* complex species.

During January 2010–March 2015, samples from case-patients in 5 hospitals affiliated with the University of São Paulo were cultured; samples positive for *C. haemulonii* were further analyzed. Clinical and epidemiologic data were retrospectively collected. Species identification of the first isolate from each patient was made by sequencing the internal transcribed spacer region of the rRNA gene (*2*). Sequence similarity searches were done by using BLAST (http://www.ncbi.nlm.nih.gov/blast). Antifungal susceptibility testing was performed by using the Clinical and Laboratory Standards Institute reference method for susceptibility testing of yeasts (*3*) for amphotericin B (AMB), fluconazole, voriconazole, caspofungin (all from Sigma, St. Louis, MO, USA), and anidulafungin (Pfizer, New York, NY, USA).

Among the 14,642 specimens that showed positive yeast cultures, 40 (0.3%) isolates from 31 patients belonged to the *C. haemulonii* complex. Most sample sources were bone and soft tissue samples from lower extremity chronic wounds (n = 17, 42%) and blood cultures (n = 11, 32%). Other positive sources were central venous catheter (CVC) tips (n = 3), toenail scrapings (n = 3), vaginal discharge (n = 2), bile (n = 1), peritoneal fluid (n = 1), pleural effusion (n = 1), and purulent fluid from the mediastinum (n = 1).

Molecular identification characterized 14 isolates as *C. haemulonii* (2 alleles), 8 as *C. haemulonii* var. *vulnera,* and 9 as *C. duobushaemulonii* (Technical Appendix Table 1). Clinical and microbiological features of the 31 patients who tested positive are summarized in the Table. Diabetes mellitus was found substantially more frequently among patients with *C. duobushaemulonii* (66% vs. 25%–28% for the other 2 species), but rates for other underlying conditions were similar for all 3 species. 

Susceptibility testing results varied by drug and species (Table). *C. duobushaemulonii* showed higher MICs for AMB than *C. haemulonii* and *C. haemulonii* var. *vulnera* , but all isolates showed high MICs for fluconazole and voriconazole. Conversely, MICs were low for caspofungin and anidulafungin. However, 1 isolate of *C. duobushaemulonii* showed high MICs of 8 μg/mL for caspofungin and 0.5 μg/mL for anidulafungin.

**Table:** Demographic, clinical, and microbiological features of patients whose cultures were positive for *C. haemulonii*. var. *vulerna*, and *C. duobushaemulonii*, January 2010–March 2015, Brazil*

Characteristic	*Candida haemulonii*, n = 14	*Candida haemulonii* var. *vulnera*, n = *8*	*Candida duobushaemulonii*, n = 9
Mean age, y (range)	42 (0–85)	46 (16–78)	49 (0–85)
Sex, F/M	8/6	6/2	4/5
Mean hospitalization, d (range)	20 (0–140)	28 (0–78)	26 (0–67)
ICU-acquired, %	2 (14)	1 (12)	4 (44)
Polymicrobial culture, %	4 (28)	5 (62)	4 (44)
Underlying conditions, %			
Malignancy†	3 (21)	3 (37)	2 (22)
Solid organ transplant	2 (14)	ND	ND
Systemic lupus erythematosus	ND	1 (12)	ND
Diabetes mellitus	4 (28)	2 (25)	6 (66)
Vascular diseases	3 (21)	3 (37)	4 (44)
Risk factors			
Previous antimicrobial drug therapy	12 (85)	6 (75)	8 (89)
Previous antifungal drug therapy	6 (42)	2 (25)	3 (33)
Chronic lower-extremity infected wounds	3 (21)	4 (50)	4 (44)
Candidemia	4 (28)	3 (37)	1 (11)
Antifungal susceptibility testing			
Amphotericin B			
GM, μg/mL (range)	1.56 (1–4)	1 (0.5–2)	4 (2–8)
MIC_90_	4	2	8
Fluconazole			
GM, μg/mL (range)	8.4 (1–64)	17.4 (2–>64)	10.07 (0.25–>64)
MIC_90_	64	64	64
Voriconazole			
GM, μg/mL (range)	1.9 (0.25–>16)	1.53 (0.125–>16)	1.07 (0.125–>16)
MIC_90_	16	16	16
Caspofungin			
GM, μg/mL (range)	0.12 (0.125–0.5)	0.26 (0.125–0.5)	0.22 (0.06–16)
MIC_90_	0.25	0.5	16
Anidulafungin			
GM, μg/mL (range)	0.015 (<0.015–0.015)	0.016 (<0.015–0.03)	0.06 (<0.015–0.5)
MIC_90_	0.015	0.03	0.5

*Values are no. (%) patients except as indicated. ND, no data; GM, geometric mean; MIC_90_, concentration that inhibits 90% of isolates.  †Solid tumors (n = 5) and hematologic malignancies (n = 3).

Of the 31 patients investigated, 11 had chronically infected wounds of lower extremities with positive surgically collected bone or soft tissue cultures, or both (Table). Samples for 4 of those patients had positive cultures for *C. haemulonii*, 3 for *C. haemulonii var. vulnera*, and 4 for *C. duobushaemulonii.* In most patients (n = 9, 82%), samples showed polymicrobial growth; *Staphylococcus* spp. (n = 7) were the most common concomitant microorganisms. All patients were treated by surgical debridement.

Samples from 8 (25%) of the 31 patients were positive for candidemia; 7 had *C. haemulonii* (3 var. *vulvera*) and 1 *C. duobushaemulonii* (Technical Appendix Table 2). Five (62%) patients had received antimicrobial drugs before the infection. Drug therapy failed in 5 (62%) that had positive cultures during deoxycholate AMB (n = 4) or fluconazole (n = 1) therapy. Among the 7 patients with CVC-associated candidemia, 4 had the CVC removed; 3 of those survived. The 30-day all-cause mortality rate was 50%.

Our study showed a prevalence of 0.3% *C. haemulonii* among yeast isolates, which was much higher than previously reported (*4*). Older commercial methods are unable to correctly identify *C. haemulonii* species, contributing to this underestimation (*4*). More closely related species such as *C. auris*, mainly found in South Africa, Asia, and the Middle East, have been misidentified as *C. haemulonii* and *C. famata* by using older systems. Thus, matrix-assisted laser desorption/ionization–time of flight mass spectrometry and internal transcribed spacer rRNA sequencing are necessary to provide the correct identification (*5*–*7*).

The data we document suggest that patients with diabetes mellitus are more likely to have positive cultures for *C. duobushaemulonii* than for the 2 *C. haemulonii* species. Moreover, *C. duobushaemulonii* isolates have higher AMB MICs than the *C. haemulonii* species. As previously reported (*8*), echinocandins showed better in vitro activity than azole compounds.

In summary, we demonstrated that *C. haemulonii* species complex are critical pathogens of chronic lower extremity wounds and that fungemia by such species remains a rare event. The 30–day all-cause mortality rate among patients with candidemia was 50%, lower than previously reported in our institution (*9*) and other centers in Brazil (*10*). We believe that in cases of candidemia by *C. haemulonii* spp. that 1) empirical use of AMB or azole compounds should be avoided; 2) removal of CVC should be performed; and 3) antifungal susceptibility testing should be done to guide antifungal therapy.

**Technical Appendix.** Molecular identification of 14 *Candida* isolates; demographic, clinical, and microbiologic characteristics of 8 cases of candidemia caused by *C.* *haemulonii* complex species; and a phylogenetic tree built with the UPGMA method by using ITS sequences of 31 clinical isolates belonging to C. *haemulonii* complex.
