# Supplementary material for: Candida haemulonii Complex Species, Brazil, January 2010–March 2015
Source: Emerg Infect Dis. 2016 Mar;22(3):561–3. doi: 10.3201/eid2203.151610 (PMC4766918; doi:10.3201/eid2203.151610)
Supplement: Supplementary file 1 — Technical Appendix. Molecular identification of 14 Candida isolates; demographic, clinical, and microbiologic characteristics of 8 cases of candidemia caused by C. haemulonii complex species; and a phylogenetic tree built with the UPGMA method by using ITS sequences of 31 clinical isolates belonging to C. haemulonii complex. [file 15-1610-Techapp-s1.pdf]

Article DOI: http://dx.doi.org.10.3201/eid2203.151610

Novel Pathogens in *Candida haemulonii* complex species, January 2010–March 2015, Brazil

Technical Appendix

Technical Appendix Table 1. Isolate name, GenBank accession number, species identification, percentage similarity to the GenBank sequences, hospital of origin, source, concomitant microrganisms, and antifungal susceptibility testing from 31 case-patients analyzed in this study*

Isolate	GenBank acession no.	Species	% Similarity (GenBank accession no.)	Hospital*	Source	Concomitant microrganisms	Antifungal susceptibility testing (Minimal inhibitory concentration μg/mL)				
							AMB	FLU	VOR	CAS	ANID
HCFMUSP01	KT236448	*Candida duobushaemulonii*	100 (JX459665.1)	Central Institute	Bone (foot ulcer)	-	2	0.25	0.12	0.12	≤0.015
HCFMUSP02	KT968725	*C. duobushaemulonii*	100 (JX459665.1)	Cancer Institute	Blood	-	4	≥64	≥16	0.25	≤0.015
HCFMUSP03	KT236449	*Candida haemulonii* var. *vulnera*	100(JX459687.1)	Central Institute	Bone (foot ulcer)	*Staphylococcus epidermidis*	1	≥64	≥16	0.25	≤0.015
HCFMUSP04	KT968726	*C. duobushaemulonii*	100 (JX459665.1)	Orthopedic Institute	Bone and soft tissue (foot ulcer)	*Finegoldia magna* and *S. epidermidis*	8	4	0.5	0.12	≤0.015
HCFMUSP05	KT968713	*Candida haemulonii*	100 (JX459684.1)	Cancer Institute	Bile	*Aeromonas hydrophila* and *Staphylococcus aureus*	1	4	2	0.12	≤0.015
HCFMUSP06	KT968733	*C. haemulonii* var. *vulnera*	100(JX459687.1)	Cancer Institute	Blood	-	0.5	32	16	0.5	≤0.015
HCFMUSP07	KT968734	*C. haemulonii* var. *vulnera*	100(JX459687.1)	Children's Institute	Blood	-	1	4	1	0.5	≤0.015
HCFMUSP08	KT968735	*C. haemulonii* var. *vulnera*	100(JX459687.1)	Central Institute	Bone and soft tissue (foot ulcer)	*S. aureus*, *Escherichia coli* and *Pseudomonas aeruginosa*	1	8	0.5	0.25	0.03
HCFMUSP09	KT968714	*C. haemulonii*	99 (JX459684.1)	Central Institute	Bone (foot ulcer)	-	2	≥64	≥16	0.25	≤0.015
HCFMUSP10	KT257659	*C. haemulonii*	99 (JX459684.1)	Central Institute	Bone (foot ulcer)	-	1	16	8	0.12	≤0.015
HCFMUSP11	KT968727	*C. duobushaemulonii*	100 (JX459665.1)	Cancer Institute	CVC-tip	-	4	8	0.25	0.12	≤0.015
HCFMUSP12	KT968715	*C. haemulonii*	100 (JX459684.1)	Heart Institute	CVC-tip	-	4	≥64	≥16	0.5	≤0.015
HCFMUSP13	KT968716	*C. haemulonii*	100 (JX459684.1)	Cancer Institute	Blood	None	1	4	1	0.25	≤0.015

Isolate	GenBank acession no.	Species	% Similarity (GenBank accession no.)	Hospital*	Source	Concomitant microrganisms	Antifungal susceptibility testing (Minimal inhibitory concentration μg/mL)				
							AMB	FLU	VOR	CAS	ANID
HCFMUSP14	KT968728	*C. duobushaemulonii*	100 (JX459684.1)	Central Institute	Toenail scrapings	None	8	4	0.5	0.12	≤0.015
HCFMUSP15	KT968729	*C. duobushaemulonii*	100 (JX459665.1)	Heart Institute	Collection from mediastinum, sternum bone	None	4	32	1	0.25	≤0.015
HCFMUSP16	KT968730	*C. duobushaemulonii*	100 (JX459665.1)	Heart Institute	Pleural effusion	None	4	8	1	0.25	≤0.015
HCFMUSP17	KT968717	*C. haemulonii*	99 (JX459684.1)	Children's Institute	Blood (2 samples)	None	4	8	4	0.25	≤0.015
HCFMUSP18	KT968731	*C. duobushaemulonii*	100 (JX459665.1)	Central Institute	Bone and soft tissue (foot ulcer)	*Serratia liquefaciens* and *Enterocccus faecalis*	8	4	0.5	8	0.5
HCFMUSP19	KT968718	*C. haemulonii*	100 (JX459684.1)	Children's Institute	Blood (3 samples)	None	1	4	0.25	0.25	≤0.015
HCFMUSP20	KT968732	*C. duobushaemulonii*	100 (JX459665.1)	Central Institute	Soft tissue (foot ulcer)	*S. aureus*	2	≥64	≥16	0.06	0.12
HCFMUSP21	KT968719	*C. haemulonii*	100 (JX459684.1)	Central Institute	Blood	None	2	4	0.25	0.25	≤0.015
HCFMUSP22	KT968720	*C. haemulonii*	99 (JX459684.1)	Central Institute	Bone (foot ulcer)	*S. aureus* and *E. coli*	1	8	4	0.25	≤0.015
HCFMUSP23	KT968721	*C. haemulonii*	100 (JX459684.1)	Central Institute	Vaginal discharge	None	4	16	1	0.12	≤0.015
HCFMUSP24	KT968736	*C. haemulonii* var. *vulnera*	100 (JX459687.1)	Orthopedic Institute	Bone and soft tissue (foot ulcer)	*Candida albicans*	2	2	0.12	0.12	≤0.015
HCFMUSP25	KT968737	*C. haemulonii* var. *vulnera*	100 (JX459687.1)	Central Institute	Bone and soft tissue (foot ulcer)	*S. aureus*	1	8	1	0.25	≤0.015
HCFMUSP26	KT968722	*C. haemulonii*	100 (JX459684.1)	Central Institute	Peritoneal fluid	None	1	1	0.5	0.12	≤0.015
HCFMUSP27	KT257660	*C. haemulonii*	100 (JX459684.1)	Children's Institute	CVC-tip	None	2	4	0.5	0.12	≤0.015
HCFMUSP28	KT968723	*C. haemulonii*	100 (JX459684.1)	Central Institute	Toenail scrapings	None	1	32	16	0.12	≤0.015
HCFMUSP29	KT968738	*C. haemulonii* var. *vulnera*	100 (JX459687.1)	Cancer Institute	Blood	None	1	64	0.5	0.12	≤0.015
HCFMUSP30	KT968724	*C. haemulonii*	100 (JX459684.1)	Central Institute	Vaginal discharge	*Corynebacterium* sp.	1	4	0.5	0.25	≤0.015
HCFMUSP31	KT968739	*C. haemulonii* var. *vulnera*	100 (JX459687.1)	Central Institute	Toenail scrapings	None	*1*	≥64	≥16	0.5	≤0.015

*Affiliated Hospitals to the University of São Paulo; AMB, amphotericin B; FLU, fluconazole; VOR, voriconazole; CAS, caspofungin; ANID, anidulafungin; CVC, central venous catheter.

Technical Appendix Table 2. Demographic, clinical, and microbiologic characteristics of 8 cases of candidemia caused by *Candida haemulonii* complex species*

Age, y/ sex	*Species*	Baseline disease	ATM/ ATF	CVC	Target antifungal therapy (days)	Therapy failure	CVC removal	Antifungal MIC (μg/mL)	30-d outcome
1/M	*C. haemulonii*	Short bowel syndrome	No	Yes	AMB (14)	AMB	Yes	AMB 4.0	Survived
8/M	*C. haemulonii*	Neuroblastoma, neutropenia	No	Yes	AMB (11), VOR (19)	AMB	Yes	AMB 1.0, VOR 0.25	Survived
16/F	*C. haemulonii var. vulnera*	Sickle-cell disease	No	No	No	ND	ND	ND	Survived
26/F†	*C.duobushaemulonii*	Ovarian carcinoma, neutropenia	ATM/ ATF	Yes	No	No	No	ND	Death
45/F	*C. haemulonii var. vulnera*	B cell lymphoma, neutropenia	ATM/ ATF	Yes	FLU (3), CAS (10)	AMB, FLU	Yes	AMB 1.0 FLU 64.0, CAS 0.12	Death
52/M	*C. haemulonii var. vulnera*	Esophagus carcimona	ATM/ ATF	Yes	AMB (5)	AMB	No	AMB 0.5	Death
63/F	*C. haemulonii*	Auto-immune cerebellar ataxia with anti-GAD antibodies	ATM/ ATF	Yes	AMB (30)	AMB	Yes	AMB 2.0	Survived
82/M†	*C. haemulonii*	WM	ATM/ ATF	Yes	FLU (3) CAS (3)	No	No	FLU 4.0 CAS 0.25	Death

*ATM, previous antimicrobial therapy; ATF, previous antifungal therapy; CVC, central venous catheter; MIC, minimal inhibitory concentration; AMB, deoxycholate amphotericin B; VOR, voriconazole; ND, no data; FLU, fluconazole; CAS, caspofungin; WM, Waldenstrom macroglobulinemia.
†Receiving palliative care.

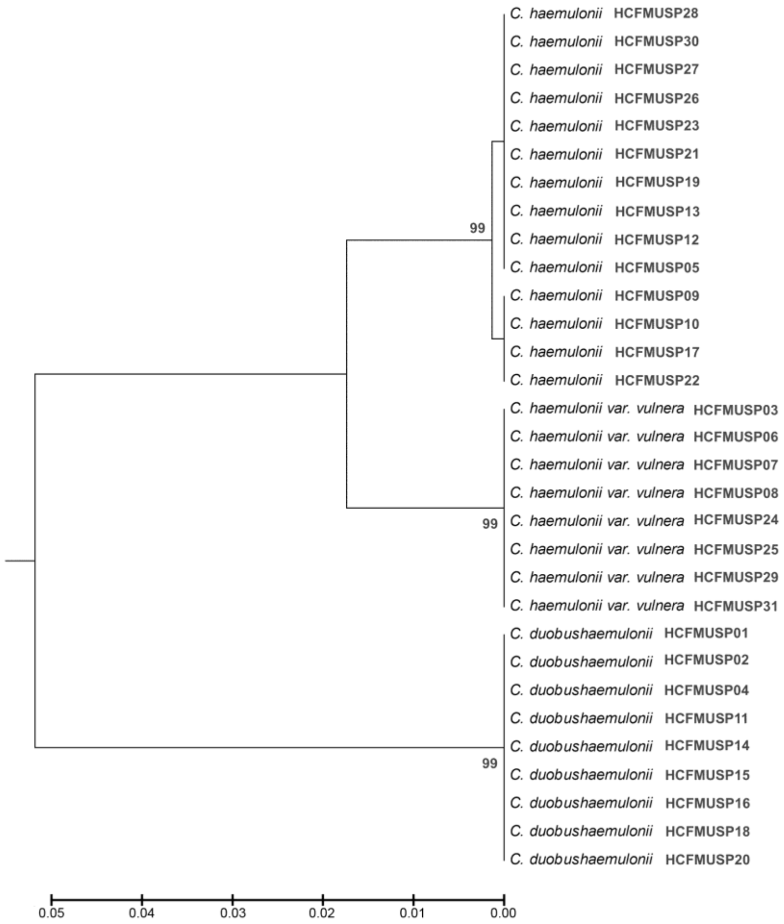

Technical Appendix Figure. Phylogenetic tree built with the UPGMA method by using ITS sequences of 31 clinical isolates belonging to *Candida haemulonii complex*. Bootstrap values are from 1,000 replicates and are given at branch nodes. Only bootstrap values >90 are indicated. The tree is drawn to scale, with branch lengths in the same units as those of the evolutionary distances used to infer the phylogenetic tree. The evolutionary distances were computed by using the Maximum Composite Likelihood method and are in the units of the number of base substitutions per site. There were a total of 376 positions in the final dataset.
